# Detection of PD-L1 in the urine of patients with urothelial carcinoma of the bladder

**DOI:** 10.1038/s41598-021-93754-z

**Published:** 2021-07-09

**Authors:** Georgi Tosev, Wasilijiang Wahafu, Philipp Reimold, Ivan Damgov, Constantin Schwab, Cem Aksoy, Adam Kaczorowski, Albrecht Stenzinger, Joanne Nyarangi-Dix, Markus Hohenfellner, Stefan Duensing

**Affiliations:** 1 Department of Urology, University Hospital Heidelberg, Im Neuenheimer Feld 110, 69120 Heidelberg, Germany; 2 Department of Urology, National Cancer Center/National Clinical Research Center for Cancer/Cancer Hospital, Chinese Academy of Medical Sciences and Peking Union Medical College, Beijing, 100020 China; 3 Division of Pediatric Nephrology, Center for Pediatric and Adolescent Medicine, University of Heidelberg, Heidelberg, Germany; 4 Institute of Medical Biometry and Informatics, University of Heidelberg, Heidelberg, Germany; 5 Department of General Pathology, Institute of Pathology, Heidelberg University Hospital, Heidelberg, Germany; 6 Molecular Urooncology, University Hospital Heidelberg, Im Neuenheimer Feld 517, 69120 Heidelberg, Germany

**Keywords:** Urological cancer, Bladder cancer

## Abstract

There are currently five programmed death-1 (PD-1)/programmed death ligand-1 (PD-L1) inhibitors approved for the treatment of locally advanced or metastatic urothelial carcinoma (UC) of the bladder. For platinum-ineligible patients, testing of tumor specimens for PD-L1 expression is required. However, scoring of PD-L1 immunohistochemistry is complex due to different antibodies used, the requirement to score expression in different cellular compartments and intratumoral heterogeneity. It can also be difficult to obtain and test longitudinal tumor samples, which would be desirable to monitor treatment responses and tumor evolution under treatment-induced selective pressure. In the present proof-of concept study, we provide evidence that PD-L1 can be detected in the urine of patients with non-muscle invasive bladder cancer (NMIBC) and muscle-invasive bladder cancer (MIBC). Urine PD-L1 levels were significantly higher in NMIBC and MIBC patients when compared to patients with various non-malignant urological diseases. Further prospective and independent studies are required to assess the value of PD-L1 in the urine as a novel biomarker with potential for the early detection, prediction and therapeutic monitoring of patients with UC of the bladder.

## Introduction

Urothelial cancer (UC) of the bladder is a heterogeneous disease with complex diagnostic, therapeutic and prognostic challenges^1^. Approximately 550,000 new patients with UC of the bladder are estimated worldwide in 2018 with almost 200,000 deaths^2^. Most patients with bladder cancer are diagnosed during diagnostic testing triggered by hematuria. Visible hematuria was found to be associated with higher-stage disease compared with non-visible hematuria^3^. The present gold standard of detection is cystoscopy with urine cytology, with sensitivity range of 68.3–100% and specificity between 57 and 97%^4^. Due to the invasiveness of the cystoscopy procedure, different urinary markers have been proposed, however, none of these markers was able to replace cystoscopy^1^. Furthermore, urine cytology, the most widely used non-invasive test to detect bladder cancer (BCa) suffers from a poor sensitivity especially for low-grade tumors^5^. Hence, current methods for both detection as well as surveillance of bladder cancer are likely to benefit from more reliable non-invasive biomarkers. Because of the ease of sampling, urinary biomarkers have attracted significant attention^5^.

PD-L1 is a transmembrane protein that plays a major role in the suppression of antitumoral T cell responses^6^. PD-L1 is widely expressed in tumor and tumor-infiltrating immune cells in patients with BCa^7^. PD-L1 expression has been found to be increased in locally advanced BCa^8,9^. Baseline tumor PD-L1 expression predicts an unfavorable response to Bacillus Calmette-Guerin (BCG) among patients with NMIBC^10^. Importantly, antibodies engineered to block PD-L1 or PD-1 have demonstrated therapeutic efficacy in patients with locally advanced or metastatic BCa^11^ leading to currently five PD-1/PD-L1 inhibitors approved for these patients^12^. Two of these five PD-1/PD-L1 inhibitors, pembrolizumab and atezolizumab, are approved as first line agents in platinum-ineligible patients but require PD-L1 testing of the tumor tissue. However, the immunohistochemical assessment of the PD-L1 status is challenging due to different antibodies approved as companion diagnostics, different scoring algorithms and intratumoral heterogeneity^13^.

In the present proof-of concept study, we analyzed whether PD-L1 can be detected in the urine of BCa patients to provide a simple, cost-effective and reliable biomarker to monitor and potentially help diagnose patients with BCa.

## Materials and methods

After obtaining written informed consent, 122 urine samples were collected and later investigated at the Department of Urology of the University Hospital Heidelberg. All urine samples were stored at room temperature using the Urine Collection and Preservation Tube 15 cc from Norgen Biotek Corp. (Cat. 18,120). Whole urine without centrifugation was used and tumor and control samples were stored for the same length of time. For the ELISA, 100 µl of urine was used and experiments were performed in duplicates. Assay performance was tested by creating a standard curve for each experiment and only experiments with a linear curve were included in the data analysis. A Quantikine ELISA for Human/Cynomolgus Monkey PD-L1/B7-H1 Immunoassay from R&D Systems was used (Catalog Number DB7H10) to measure PD-L1 in the urine samples. This study was approved by the Ethics Committee of the University of Heidelberg (F-760/2019). Informed consent is taken from all the participants present in the study. All reported investigations were conducted in accordance with our national regulations and according to the updated version of the Declaration of Helsinki.

Clean-voided urine samples were collected from individuals belonging to three groups (Table 1): group 1 (N = 20) were patients with newly diagnosed UC of the bladder before undergoing transurethral resection of the bladder (TURB) (before TURB group; 16 patients with NMIBC and 4 patients with MIBC), group 2 (N = 63) consisted of patients with recurrent UC of the bladder previously treated with TURB (after TURB group; 31 patients with NMBIC and 32 patients with MBIC) and a control group (group 3, N = 39) consisting of patients diagnosed with nonneoplastic diseases (Table 2). All patients with newly diagnosed BCa (group 1) had a positive cystoscopic evaluation prior to urine collection. Group 2 (after TURB) was heterogeneous. A total of 31 post-TURB samples were collected from patients treated at the University Hospital Heidelberg. In 28 of these patients, the TURB was performed six weeks to several months before sampling and patients had a positive cystoscopy before urine sample collection for the present study. In the remaining three post-TURB patients, the cystoscopy before urine sample collection was negative. In 32 patients, a TURB was performed externally and patients were seen at the University Hospital Heidelberg for a second opinion. Ten of these 32 patients were sampled 6 to 15 weeks after a TURB and a negative cystoscopy was documented before urine sample collection. In 22 of the 32 patients, no cystoscopy results were available since the TURB was performed less than four weeks before sample collection. In total, a negative cystoscopy was documented in 13 of 63 patients of the post-TURB group before collection of urine samples. Ten patients in the post-TURB group had received neoadjuvant therapy. Post-TURB patients were at different stages of their treatment with mitomycin C and/or BCG therapy and differential time since the first TURB (median time from TURB to sample collection 60 days, inter-quartile range [IQR] 24–105 days). All 63 patients in group 2 have had more than two TURBs in their medical history at the time of urine sample collection.

**Table 1:** Baseline clinical and pathological features of healthy controls and patients with bladder cancer along with urinary levels of PD-L1.

Variable	Bladder cancer						Overall			Control N = 39
	Group 1 (Before TURB)			Group 2 (After TURB)						
	NMIBC N = 16	MIBC N = 4	*p* value*	NMIBC N = 31	MIBC N = 32	*p* value**	NMIBC N = 47	MIBC N = 36	*p* value**	
Age (y)			**0.0002**			** < 0.0001**			** < 0.0001**	
Median	68.0	70.5		66.0	63.5		67.0	66.5		50.0
IQR	59–78	70–72		58–75	57–76		58–76	57–74		33–61
Sex (no., %)			0.1944			0.8175			0.4585	
Female	1 (6)	0		6 (19)	7 (22)		7 (15)	7 (19)		10 (26)
Male	15 (94)	4 (100)		25 (81)	25 (78)		40 (85)	29 (81)		29 (74)
Grade (no., %)										
Low	7 (44)	0		10 (32)	3 (9)		17 (36)	3 (8)		n.a
High	9 (56)	4 (100)		21 (68)	29 (91)		30 (64)	33 (92)		n.a
TNM-stage (no., %)										
pTa	12 (75)	0		12 (39)	0		24 (51)	0		n.a
pTis	0	0		1 (3)	0		1 (2)	0		n.a
pT1	4 (25)	0		18 (58)	0		22 (47)	0		n.a
pT2	0	3 (75)		0	28 (88)		0	31 (86)		n.a
pT3***	0	0		0	3 (9)		0	3 (8)		n.a
pT4***	0	1 (25)		0	1 (3)		0	2 (6)		n.a
PD-L1 concentration (pg/mL)			** < 0.0001**			**0.0003**			** < 0.0001**	
Median	11.28	71.73		7.90	4.10		9.55	5.93		0
IQR	0–21	27–123		0–20	0–12		0–20	0–17		0–3
Mean	11.00	75.29		18.67	33.16		16.06	37.85		1.93
Standard deviation	11.03	58.84		31.18	90.60		26.21	88.02		4.07

*Abbreviations* IQR: inter-quartile range; n.a.: not applicable; NMIBC: non-muscle invasive bladder cancer; MIBC: muscle-invasive bladder cancer; TNM: tumor, node, metastasis (classification); TURB: transurethral resection of the bladder.

*Kruskal–Wallis and Fisher’s exact test used to determine if there was significant variation in the medians of the control and NMBIC and MIBC groups for continuous (age and PD-L1 concentration) and categorical variables (sex), respectively.

**Kruskal–Wallis and Chi-Square test used to determine if there was significant variation in the medians of the control and NMBIC and MIBC groups for continuous (age and PD-L1 concentration) and categorical variables (sex), respectively.

***The final pathological stage was determined after radical cystectomy following urine sample collection.

**Table 2:** Control group according to nonneoplastic diagnosis.

Nonneoplastic diagnosis	Concentration (pg/mL)		
	No. of patients	Median	IQR
Benign prostatic hyperplasia	3	0	0–4.30
Bladder outlet obstruction	1	0	0–0.00
Chronic urocytitis	3	0	0–0.00
End-stage renal disease and urocystitis follicularis	1	0	0–0.00
Epididymitis	1	0	0–0.00
Erectile dysfunction	1	2.95	2.95–2.95
Hematospermia	1	0	0–0.00
Hematuria	1	4.45	4.45–4.45
Hydrocele testis	1	0.45	0.45–0.45
Pelvic-ureteric junction obstruction	1	0	0–0.00
Peyronie's disease	1	6	6.00–6.00
Pyelonephtis	2	0	0–0.00
Renal calculi	17	0	0–2.95
Urinary tract infection	2	0	0–0.00
Varicocele	1	0	0–0.00
Other*	2	0	0–0.00

*Abbreviations* IQR: interquartile range.

*Consists of 2 patients with dilated cardiomyopathy.

Demographic data and pathological features were presented for control patients and summarized according to NMIBC vs MIBC type for groups 1 and 2, whereby categorical variables are presented as frequency distributions. Median and IQR were reported for continuous variables. For statistical tests, *p* < 0.05 was considered significant. Statistical analysis was performed using SAS software, version 9.4 (SAS Institute Inc., Cary, N.C., USA).

### Informed consent

Informed consent is taken from all the participants present in the study.

### Ethical approval

This study was approved by the ethics committee of the University of Heidelberg (F-760/2019).

## Results

**Figure 1:**
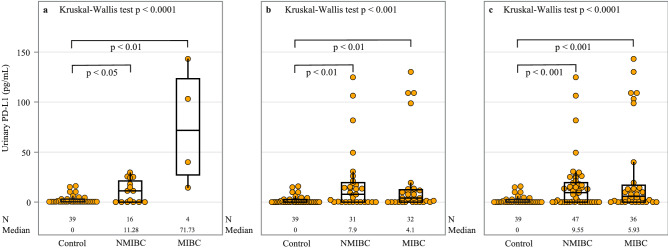
Distribution of PD-L1 concentration in controls and NMIBC and MIBC sub-groups. Patients with BCa are sampled from: (**a**) group 1 (before TURB), (**b**) group 2 (after TURB) and (**c**) combined groups 1 and 2. Results are presented by a box plot, with each dot representing one patient. An extreme outlier of 487.5 pg/mL in the MIBC sub-group in (**b**) and (**c**) is not shown on the plot. Analysis of urinary PD-L1 concentrations demonstrated departure from Gaussian distribution using Shapiro–Wilk test in all groups. Non-parametric Kruskal–Wallis test was thus used to determine if there was significant variation in the medians of the groups analyzed. If significant at the 5% level, we then used Dunn's multiple comparison post-hoc test to investigate pair-wise group comparisons of urinary PD-L1. This testing procedure was followed in (**a–c**). Only significant comparisons as follows from Dunn's post-hoc test are shown. Abbreviations: BCa: bladder cancer; N: number of observations; NMIBC: non-muscle invasive bladder cancer; MIBC: muscle-invasive bladder cancer; PD-L1: programmed death ligand-1; TURB: transurethral resection of the bladder.

A summary of PD-L1 urinary expression is shown in Table 1 along with demographic data and pathological features. Groups differed significantly according to age, but not by sex of the patients. Figure 1 presents the distribution of PD-L1 concentration in controls and patients with NMIBC and MIBC. The Kruskal–Wallis test showed that there was significant variation in the medians of the groups analyzed (*p* < 0.001 to *p* < 0.0001), whereby Dunn's multiple comparison post-hoc was then applied between groups. Median urine levels of PD-L1 were elevated in both newly diagnosed (11.28 and 71.73 pg/mL in NMBIC and MIBC subgroups, with an IQR of 0–21 and 27–123, respectively) and recurrent bladder cancer patients (7.9 and 4.1 pg/mL in NMBIC and MIBC sub-groups, with IQR of 0–20 and 0–12, respectively) in comparison to the control group (0 pg/mL, IQR: 0–3) with *p* < 0.05 to *p* < 0.01 (Fig. 1 A and B). In the post-TURB group (group 2), a direct comparison of the PD-L1 urine level between patients with a documented negative cystoscopy (N = 13) and the other post-TURB patients (N = 50) showed no statistically significant differences (median 4.45 pg/ml, IQR 1–14 pg/ml versus 5.93 pg/ml, IQR, 0–20 pg/ml; *p* = 0.99).

**Figure 2:**
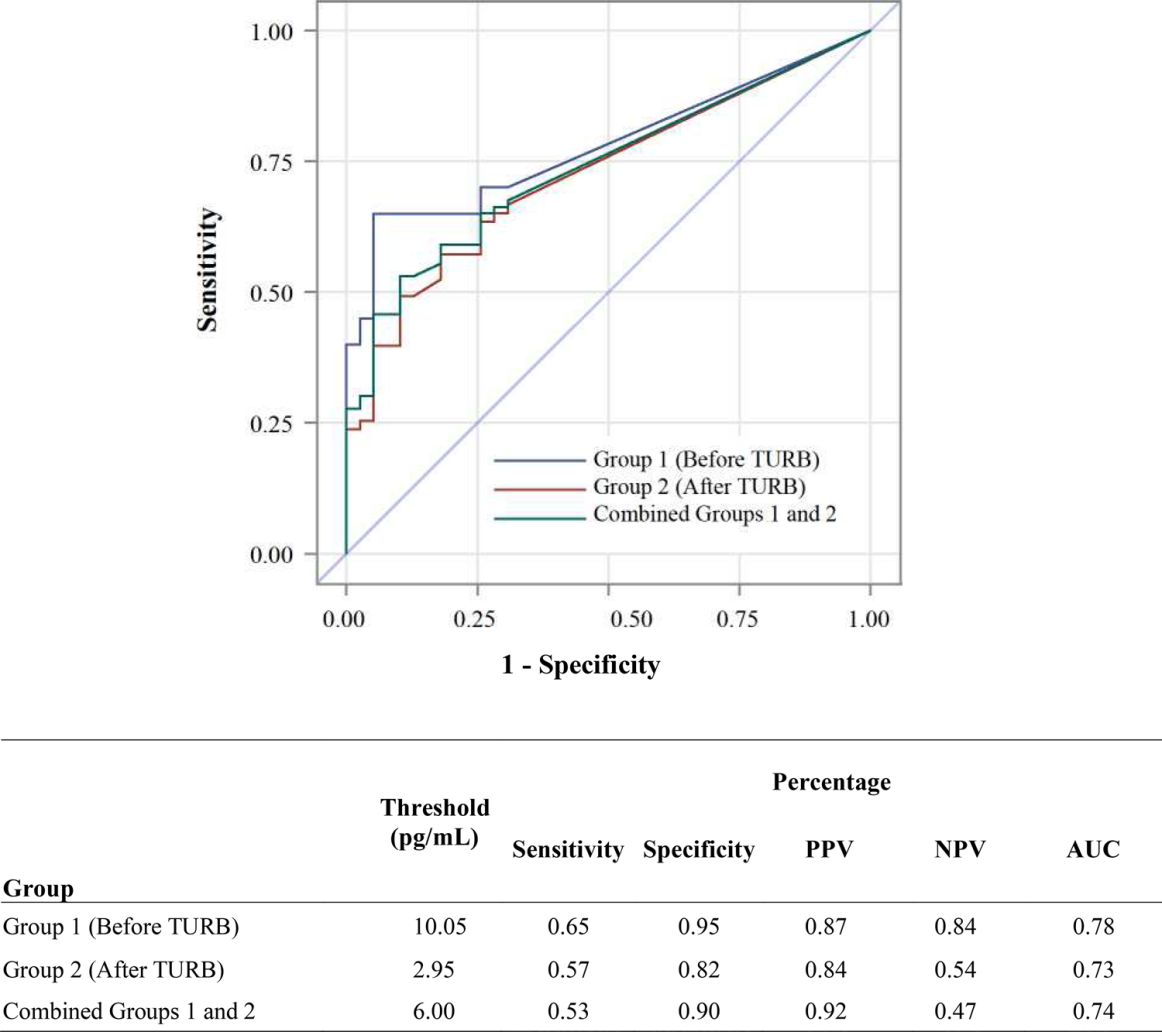
Diagnostic profile of urinary PD-L1 using ROC analysis. Abbreviations: AUC: area under the curve; NPV: negative predictive value; PPV: positive predictive value; TURB: transurethral resection of the bladder.

The urine PD-L1 expression in the control group is summarized in Table 2. In addition, Fig. 2 presents the diagnostic profile of urinary PD-L1 using receiver operating characteristic (ROC) curve analysis, whereby the Youden index was used to determine the optimal cutoff point. The threshold PD-L1 concentration in pg/mL was calculated as 10.05 and 2.95 for comparison between controls and newly diagnosed pre-TURB patients in group 1 and post-TURB patients in group 2, respectively. Area under the ROC curve (AUC), an accuracy index for evaluating the predictive performance, was highest (0.78) when used for detection of newly diagnosed BCa patients (group 1), with sensitivity and specificity of 0.65 and 0.95 and positive and negative predictive values of 0.87 and 0.84. These initial metrics values compare well with published results for urinary protein biomarker tests approved by the U.S. Food and Drug Administration (FDA)^5^. While no single protein biomarker has yet achieved the desired accuracy, PD-L1 could potentially become a valuable biomarker as addition to a multiparametric panel for the monitoring and potentially diagnosis of BCa.

## Discussion

This proof-of-concept study shows that elevated urinary PD-L1 levels are present in both newly diagnosed NMIBC and MIBC patients prior to treatment with TURB as well as post-TURB NMIBC and MIBC cases as compared with control subjects diagnosed with nonneoplastic diseases. Of note, in this pilot study, we did not implement inclusion or exclusion criteria when enrolling patients resulting in a heterogenous group of BCa patients. Furthermore, the calculation of predictive accuracy is of limited evidence since the diagnosis was known at the time of urine collection.

Our results raise a number of important questions. First, what is the source of PD-L1 in the urine of BCa patients? To address this point, we prospectively collected urine from nine patients with urothelial carcinoma of the bladder and analyzed the specimens for urine PD-L1 levels, urine cytology and PD-L1 protein expression in urine exosomes (Supplementary Information, Fig. S1). We found no signs of acute inflammation in the urine cytology*.* One of the nine patients showed a urine cytology that was positive for malignant cells but PD-L1 was undetectable in the urine of this patient suggesting that sources other than cancer cells may be involved in urine PD-L1 levels. We detected significant exosomal PD-L1 protein by immunoblotting in three of the nine patient samples analyzed. However, there was no correlation between exosomal PD-L1 expression and urine PD-L1 levels. Taken together, these results suggest that other sources than acute inflammation or cancer cells may lead to increased PD-L1 levels in the urine of patients with BCa. Remarkably, a recent study by Alanee and colleagues found a significant increase of PD-L1-positive white blood cells, predominantly CD4-positive lymphocytes, in the urine of BCa patients^14^. Moreover, Chevalier and colleagues discovered an expansion of a newly identified PD-L1-positive, CD4-positive T regulatory cell population^15^ in the urine of BCa patients, interestingly without a corresponding increase of this immune cell population in the peripheral blood of these patients^16^. It is hence conceivable that urine PD-L1 expression may stem from PD-L1 positive immune cells. The dynamics of PD-L1 expression in the urine after tumor removal also requires further investigation.

A second question pertains to the relationship between urine and tissue PD-L1 expression. To address this question, we performed an analysis of 13 patients taken of our study with PD-L1 urine levels ranging from 0 to 487 pg/ml for tissue PD-L1 expression (Supplementary Information, Fig. S2). Three immunohistochemical PD-L1 staining scores were calculated—(1) Tumor Proportion Score, TPS, i.e., the percentage of viable tumor cells presenting with membranous PD-L1 staining of any intensity, (2) Immune Cell Score, ICS, i.e., tumor-infiltrating immune cells positive for PD-L1 occupying a certain proportion of the tumor area and (3) Combined Positivity Score, CPS, i.e., positively stained tumor cells and tumor-infiltrating lymphocytes and macrophages divided by the total number of viable tumor cells multiplied by 100*.* A weak positive correlation between PD-L1 urine levels and tissue PD-L1 scores of 0.29 was found only for the ICS but not for the TPS or CPS (correlation coefficients − 0.27 and − 0.24, respectively; Supplementary Information, Fig. S2. These results suggest that immune cells may play a role in urine PD-L1 expression in line with previous studies^14,16^. However, since secreted forms of PD-L1 have been reported^17^, our results cannot exclude that this source of urine PD-L1 contributes to our findings.

Our study is, to the best of our knowledge, the first to use an ELISA-based method to detect PD-L1 in the urine of BCa patients. Other studies have used flow cytometry in BCa patients^14,16^ or urine mRNA expression, albeit under different disease conditions^18,19^. Further prospective and independent evaluations, in particular longitudinal studies, are required to assess urinary PD-L1 as a biomarker for the monitoring and detection of BCa, building upon the initial evidence we present here.

## Supplementary Information

Supplementary Information.
