# Publisher Correction: Detection of PD-L1 in the urine of patients with urothelial carcinoma of the bladder

**DOI:** 10.1038/s41598-021-97827-x

**Published:** 2021-09-09

**Authors:** Georgi Tosev, Wasilijiang Wahafu, Philipp Reimold, Ivan Damgov, Constantin Schwab, Cem Aksoy, Adam Kaczorowski, Albrecht Stenzinger, Joanne Nyarangi‑Dix, Markus Hohenfellner, Stefan Duensing

**Affiliations:** 1 Department of Urology, University Hospital Heidelberg, Im Neuenheimer Feld 110, 69120 Heidelberg, Germany; 2 Department of Urology, National Cancer Center/National Clinical Research Center for Cancer/Cancer Hospital, Chinese Academy of Medical Sciences and Peking Union Medical College, Beijing, 100020 China; 3 Division of Pediatric Nephrology, Center for Pediatric and Adolescent Medicine, University of Heidelberg, Heidelberg, Germany; 4 Institute of Medical Biometry and Informatics, University of Heidelberg, Heidelberg, Germany; 5 Department of General Pathology, Institute of Pathology, Heidelberg University Hospital, Heidelberg, Germany; 6 Molecular Urooncology, University Hospital Heidelberg, Im Neuenheimer Feld 517, 69120 Heidelberg, Germany

Correction to: *Scientific Reports* 10.1038/s41598-021-93754-z, published online 09 July 2021

The original version of this Article contained errors in Figure 1, where the horizontal bars indicating between groups comparison were omitted.

The original Figure 1 and accompanying legend appear below.

**Figure 1:**
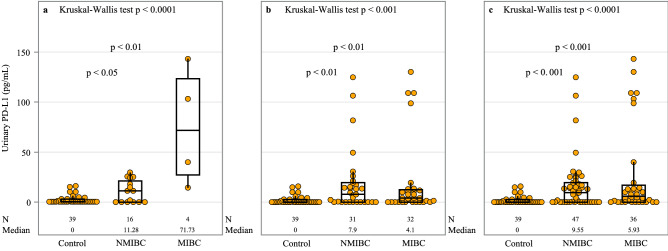
Distribution of PD-L1 concentration in controls and NMIBC and MIBC sub-groups. Patients with BCa are sampled from: (**a**) group 1 (before TURB), (**b**) group 2 (after TURB) and (**c**) combined groups 1 and 2. Results are presented by a box plot, with each dot representing one patient. An extreme outlier of 487.5 pg/mL in the MIBC sub-group in (**b**) and (**c**) is not shown on the plot. Analysis of urinary PD-L1 concentrations demonstrated departure from Gaussian distribution using Shapiro–Wilk test in all groups. Non-parametric Kruskal–Wallis test was thus used to determine if there was significant variation in the medians of the groups analyzed. If significant at the 5% level, we then used Dunn's multiple comparison post-hoc test to investigate pair-wise group comparisons of urinary PD-L1. This testing procedure was followed in (**a–c**). Only significant comparisons as follows from Dunn's post-hoc test are shown. Abbreviations: BCa: bladder cancer; N: number of observations; NMIBC: non-muscle invasive bladder cancer; MIBC: muscle-invasive bladder cancer; PD-L1: programmed death ligand-1; TURB: transurethral resection of the bladder.

The original Article has been corrected.

