# Supplementary material for: Detection of PD-L1 in the urine of patients with urothelial carcinoma of the bladder
Source: Sci Rep. 2021 Jul 9;11:14244. doi: 10.1038/s41598-021-93754-z (PMC8270894; doi:10.1038/s41598-021-93754-z)
Supplement: Supplementary file 1 — Supplementary Information. [file 41598_2021_93754_MOESM1_ESM.docx]

**Supplementary information**

**Detection of PD-L1 in the Urine of Patients with Urothelial Carcinoma of the Bladder**

**Georgi Tosev^1*^, Wasilijiang Wahafu^1,2*^, Philipp Reimold^1^, Ivan Damgov^3,4^, Constantin Schwab^5^, Cem Aksoy^1^, Adam Kaczorowski^6^, Albrecht Stenzinger^5^, Joanne Nyarangi-Dix^1^, Markus Hohenfellner^1^, Stefan Duensing^1,6^**

^1^Department of Urology, University Hospital Heidelberg, Im Neuenheimer Feld 420, D-69120, Heidelberg, Germany.

^2^Department of Urology, National Cancer Center/National Clinical Research Center for Cancer/Cancer Hospital, Chinese Academy of Medical Sciences and Peking Union Medical College, 100020, Beijing, China.

^3^Division of Pediatric Nephrology, Center for Pediatric and Adolescent Medicine, Im Neuenheimer Feld 430, University of Heidelberg, D-69120 Heidelberg, Germany.

^4^Institute of Medical Biometry and Informatics, University of Heidelberg, Im Neuenheimer Feld 130, D-69120 Heidelberg, Germany.

^5^Institute of Pathology, Heidelberg University Hospital, Im Neuenheimer Feld 224, D-69120 Heidelberg, Germany.

^6^Molecular Urooncology, University Hospital Heidelberg, Im Neuenheimer Feld 517, D-69120, Heidelberg, Germany.

**Figure S1.** Urine levels of PD-L1, urine cytology and exosomal PD-L1 expression were measured in nine patients with urothelial carcinoma of the bladder. Exosomes were isolated using a commercially available kit and an immunoblot for PD-L1 protein expression is shown. An immunoblot for TSG101 protein is shown as a urine exosome marker. Abbreviations: PD-L1: programmed death ligand-1; TSG101: tumor susceptibility gene 101.

**
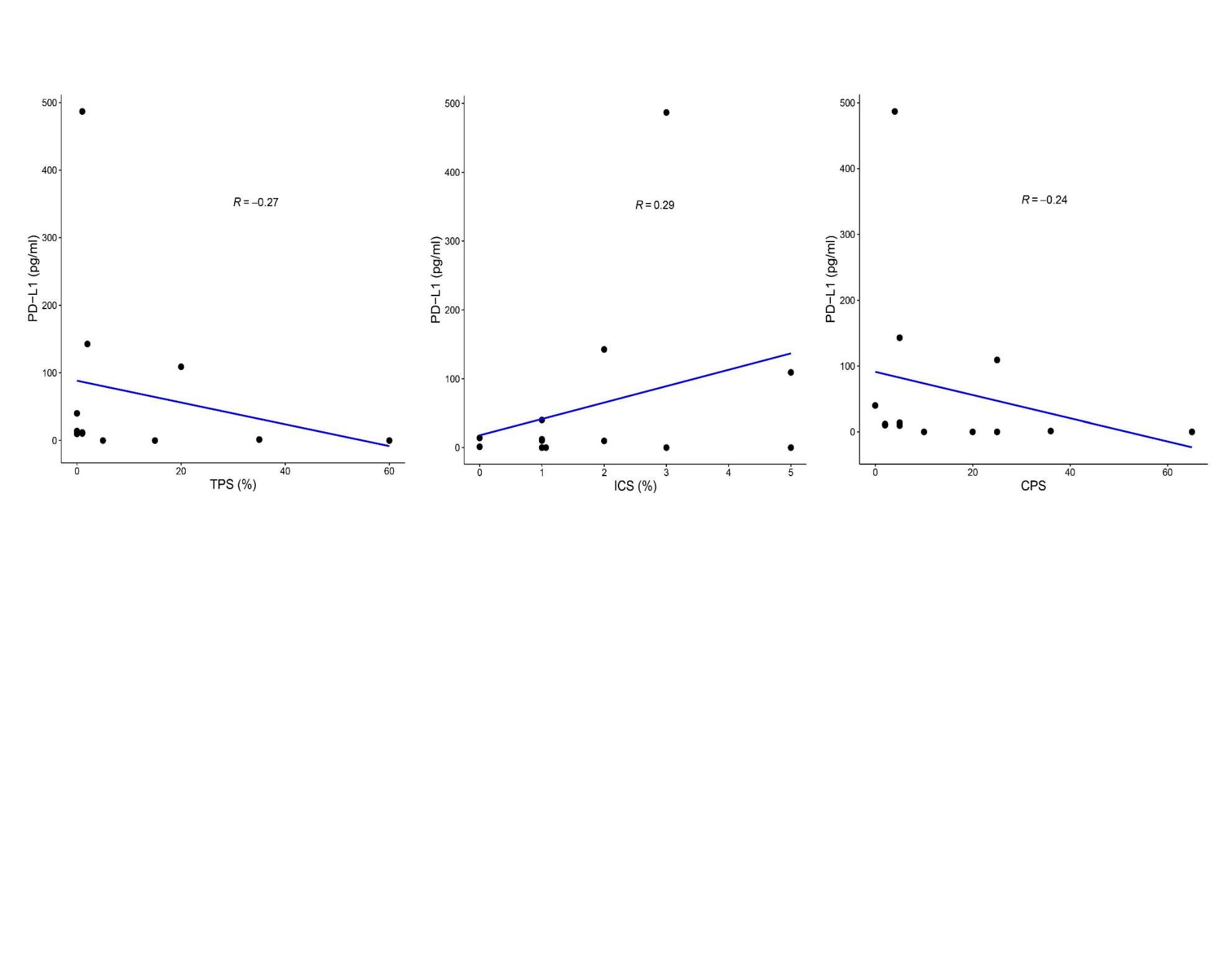
**

**Figure S2.** Correlation between urine PD-L1 levels and tissue PD-L1 immunohistochemistry scores. Blue line depicts line of best fit of a univariable linear regression. Abbreviations: CPS: combined positivity score; ICS: immune cell score; PD-L1: programmed death ligand-1; *R:* Pearson’s correlation coefficient; TPS: tumor proportion score.

References

1. Pisitkun, T., Shen, R.-F. & Knepper, M. A. Identification and proteomic profiling of exosomes in human urine. *Proceedings of the National Academy of Sciences of the United States of America* **101,** 13368–13373 (2004).
